# Neural representations of the value of helping the family during adolescence

**DOI:** 10.1016/j.dcn.2026.101770

**Published:** 2026-06-27

**Authors:** Jasmine Hernández, Jessica Uy, Naomi I. Eisenberger, Adriana Galván, Eva H. Telzer, Andrew J. Fuligni

**Affiliations:** a University of California Los Angeles, Los Angeles, CA, United States; b Stanford University, Stanford, CA, United States; c UNC-Chapel Hill, Chapel Hill, NC, United States

**Keywords:** Family obligation values, Prosocial behavior, FMRI, Brain development, Adolescence

## Abstract

Supporting one’s family is an important value for many adolescents across diverse cultural backgrounds. As a developmental period characterized by increased value-based decision-making and ongoing changes in the developing brain, adolescence presents a valuable opportunity to investigate the neural representations of this value. The present study examined the association between adolescents’ sense of obligation to the family and functional connectivity between neural systems involved in motivation and reward, social cognition, and cognitive control when making decisions about whether to provide financial support to the family at a cost to themselves. Participants included 266 adolescents (ages 9–15 and 19–20) who completed a functional magnetic resonance imaging (fMRI) decision-making task in which they had the opportunity to give money to caregivers, friends, and strangers. Family obligation values consistently predicted negative functional connectivity between the ventral striatum (VS) and networks associated with social cognition and cognitive control during decisions to give to the family among older adolescents, but this association was connectivity-dependent (reduced and sometimes positive) among younger youth. As such, regions previously implicated in mentalizing and cognitive control may modulate the relative weight placed on family obligation values during value-based functional coupling in the VS. These findings reflect the importance of corticostriatal connectivity in integrating the relative value of helping the family in promoting prosocial behavior during adolescent development.

## Highlights

• In older adolescents, familial values predicted negative connectivity between key networks.
• This association was connectivity-dependent among younger adolescents.
• Connectivity may elucidate complexity in prosocial decision-making across development.

## Graphical Abstract

**Figure:**
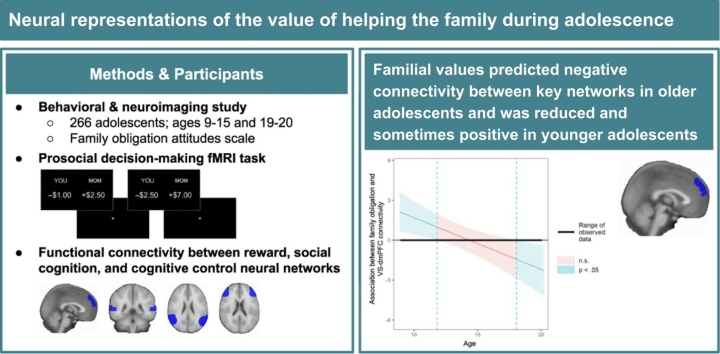

## 1 Introduction

The value placed on supporting and assisting the family is important for many adolescents across diverse cultural backgrounds. The importance of this value, often referred to as family obligation, may shape motivation, behavior, and adjustment across adolescence, even for youth from cultural backgrounds who do not traditionally place a strong emphasis on them (Fuligni et al., 1999, Fuligni and Pedersen, 2002, Suarez-Orozco and Suarez-Orozco, 1995). As a developmental period underpinned by increased value-based decision-making and ongoing changes in the developing brain, adolescence may present an opportunity to investigate the neural representations associated with this value (Davidow et al., 2018, Pfeifer and Berkman, 2018). This study makes novel contributions through examining how the dynamic integration and functional connectivity between interacting neural systems during decisions to assist others (e.g., engage in prosocial behaviors) may provide important insight into age-related differences in value-based integration (van Hoorn et al., 2019). The present study examined the association between the value adolescents placed on helping the family and the functional connectivity between systems involved in motivation and reward, social cognition, and cognitive control when making decisions about whether to provide financial support to their family at a cost to themselves across a broad age range of youth.

### 1.1 Value of family assistance

The value of helping the family may provide many youth with a meaningful social role and reflect a key developmental process of social identity development. This value is reflected through adolescents’ engagement in prosocial behaviors to help the family, which can include providing instrumental support, such as completing household chores (e.g., cleaning, cooking), caring for siblings, and assisting parents at their work (e.g., completing government forms; Fuligni et al., 1999, Suarez-Orozco and Suarez-Orozco, 1995). Consistent with social identity theory, group identification enhances one’s willingness to support and assist one’s group and provides a sense of meaning and purpose (Hogg, 2003), which is linked to better psychological well-being (Zika and Chamberlain, 1992). Thus, these values may function as developmental assets that may shape adolescents' social identities. Family obligation values are also associated with a range of positive developmental outcomes during adolescence, including heightened academic motivation (Fuligni, 2001), improved school adjustment (van Geel and Vedder, 2011), enhanced psychological well-being (Fuligni et al., 2002), and reduced engagement in substance use and risky behaviors (Telzer et al., 2014, Telzer et al., 2014). Importantly, these associations are present even among youth from groups that do not traditionally place strong emphasis on helping the family.

### 1.2 Neural representation of value

Values inherently serve as motivational constructs that may guide decision-making. An extensive body of neuroeconomic and social-cognitive research has assessed the computational and neural processes involved in value-based decision-making by measuring neural responses to value input from the environment, defined as either-or choices between two or more options with varied attributes (Lebreton et al., 2009, Levy and Glimcher, 2012, Pfeifer and Berkman, 2018, Rangel and Hare, 2010). In this approach, different value attributes are weighed and then transformed into a common neural currency (Pfeifer and Berkman, 2018). From this perspective, these attributes contribute to subjective value computation to yield decisions and may be represented in the brain in regions implicated in processing value or reward, such as the ventral striatum (VS). Across adolescence, heightened activity in the VS in response to self and other-oriented rewards plays an important role in motivating decisions, such as the rewarding experience of helping others (Moll et al., 2006, Telzer et al., 2011). As such, the VS, which codes the motivational value of a given decision, receives input from interconnected cortical regions, that support higher-order cognitive functions (e.g., mentalizing) via looped, anatomical pathways (Haber et al., 2000, Haber and Knutson, 2010). The most commonly implicated regions, which includes the dorsomedial prefrontal cortex (dmPFC), temporal parietal junction (TPJ), and posterior superior temporal sulcus (pSTS), also referred to as the social brain network, are involved in mentalizing processes, such as shifting attention towards the needs and values of others and perspective taking (Blakemore and Mills, 2014).

### 1.3 The development of the neural representation of value

Neural systems associated with motivation, reward, and social cognition also undergo significant functional maturation during adolescence. An increasing body of developmental neuroscience research has examined how these networks interact to shape value-based and goal-oriented decision-making. When making value-based decisions, it is often necessary to engage in a level of self-control to resolve conflict between prosocial motives and self-interests in order to put the needs of another before one’s own immediate needs (Fehr and Camerer, 2007). This ability to regulate one’s thoughts, feelings, and behaviors, has been consistently linked to activation in bilateral ventrolateral (vlPFC) and dorsolateral (dlPFC) prefrontal cortices (Bellucci et al., 2020, Schreuders et al., 2019, van de Groep et al., 2020). Given this, stronger functional connectivity between these regions and subcortical (e.g., VS) regions may reflect an upregulation of higher order cognitive functions that facilitates prosocial behaviors during adolescence (Gęsiarz and Crockett, 2015, Telzer et al., 2011). Importantly, the valuation process integrates signals from regions that represent relevant attributes of decisions, and especially during adolescence, this integration appears to occur in tandem with the merging of self and other—such as through mentalizing about and giving to others—via a common neural network including the medial prefrontal cortex (medial PFC) (Crone and Fuligni, 2020). Connectivity between corticostriatal systems may simultaneously support value integration and social-affective processing during other-oriented decision-making. As such, examining how these processes change throughout development could provide insight into these mechanisms (Pfeifer and Berkman, 2018).

### 1.4 Neural representation of the value of family assistance

Prior brain imaging research suggests that these neural networks are involved during prosocial decisions to help the family, all of which undergo significant functional maturation during adolescence. Studies have suggested that during adolescence, youth increasingly prioritize giving to familiar others, such as family and friends, more than strangers (van de Groep et al., 2022, Güroğlu et al., 2014, Padilla-Walker et al., 2018). At the neural level, giving resources to family has been linked with neural activation in regions implicated in cognitive control (dlPFC, vlPFC), social cognition (dmPFC, pSTS), and reward processing (VS, NAcc) (Bellucci et al., 2020, Crone and Fuligni, 2020, Eisenberger, 2013, Keltner et al., 2014, Telzer et al., 2010, Telzer et al., 2011, Telzer et al., 2013). Relatively few studies, however, have established a link between individuals’ family obligation values and associated neural processing across development. In the first and only study to date to investigate the role of family obligation values within prosocial decision-making, Telzer et al. (2011) demonstrated that individuals who value helping the family to a greater extent show greater functional coupling between the VS and regions involved in mentalizing (dmPFC) and cognitive control (dlPFC) during costly giving to the family. However, this study only considered a single age group, the average being 20 years of age.

Examining age differences is important, as adolescence is a transition period marked by the acquisition of mature social roles and goals in conjunction with age- and experience-related changes in brain circuitry. In the transition to young adulthood, individuals’ sense of familial obligation to help the family increases and its meaning may shift with age, as reflected in changes to the parent-child relationship, a deeper understanding of reasons to help the family, and thus might become more internalized or automatic as it progressively becomes a typical aspect of daily life. As such, these changes may have implications for the developmental nature of functional connectivity between these networks. It is possible that the nature of the connectivity may or may not change to support these prosocial behaviors with age. For instance, perhaps at younger ages, stronger connectivity between these regions may reflect greater coordination between reward and sociocognitive processes in order to elicit prosocial decisions, whereas at older ages, this connectivity might change to support the same behavior, which might not necessitate stronger connectivity between such networks (Blakemore, 2008, Kwon et al., 2022, Telzer et al., 2011). The ongoing refinement and connectivity between neural systems involved in social cognition, cognitive control, and reward processing across development may thus elucidate the complexity involved in how the value placed on family obligation influences adolescents’ decisions to help others across development. Taken together, these findings highlight the added value of investigating the interaction between family obligation values and functional connectivity across different social contexts, such as when the target recipient is a friend or stranger, across adolescence.

### 1.5 Current study

This study investigated the association between the value adolescents placed on helping the family and the functional connectivity between neural systems implicated in motivation and reward, social cognition, and cognitive control. Participants across a broad age range during adolescence (9–20 years) performed a functional magnetic resonance imaging (fMRI) prosocial decision-making task in which they made decisions whether to provide financial support to their family at a cost to themselves. We hypothesized that adolescents who placed greater value on helping the family would exhibit stronger functional connectivity between the VS and regions involved in social cognition and cognitive control than adolescents who value helping the family less. We also examined whether these neural patterns were family-specific or extend to prosocial decisions involving others, such as friends or strangers, and whether these associations differed across age. Some scholars have proposed that these values inherently orient youth towards prosociality across different social domains because they guide the youth in being considerate of others more broadly (Carlo et al., 2022). At the same time, a social identity perspective might predict that valuing family assistance would predict neural connectivity only during giving to the family and not to friends and strangers, given that the values are specific to the in-group of the family and not other groups (Fuligni and Flook, 2005). Thus, we predicted increased differentiated functional connectivity according to target between regions implicated in cognitive control, social cognition, and motivation and reward when giving to the family in comparison to friends and strangers. Whether this functional coupling is greater with age remains an open question, but it’s possible that connectivity in these circuits changes developmentally to support other-oriented decision-making as suggested in prior literature (Kwon et al., 2022). On one hand, the subjective value of helping the family might increase with age given the increased specialization and connectivity between these functional networks across development. On the other hand, in this particular context of the value and decision to give to the family, youth may also increasingly gain a more sophisticated understanding of what it means to have an obligation to the family, which may yield differentiated connectivity between value-based and prefrontal regions with age.

## 2 Methods

### 2.1 Participants

Adolescents aged 9–15 years were recruited through flyers, advertisements, and classroom presentations at schools within the Los Angeles Unified School District. Additional participants were identified through the Clinical and Translational Science Institute database, which includes families affiliated with the University of California, Los Angeles (UCLA) and its medical systems. Finally, older adolescents (ages 19–20) were recruited from UCLA undergraduate classes. All participants were fluent in English, right-handed, had no MRI contraindications or prior psychiatric diagnoses, and were not pregnant or attempting to become pregnant at the time of the study. Written consent and assent were obtained from parents and youth in accordance with UCLA's Office of the Human Research Protection Program and institutional review board.

A total of 266 participants (ages 9–15 [n = 227], 19–20 [n = 39]) participated in the study by completing the survey measures and/or participating in the fMRI scan session. The final sample for the present study included 210 participants who completed relevant survey measures and participated in the scan session, after applying exclusions if more than 20% of frames had a framewise displacement exceeding 0.9 mm and had good quality scans. Participants were recruited from two studies: approximately half (n = 129) participated in a cross-sectional study (Study 1) and the remainder (n = 137) were part of the first wave of a longitudinal study (Study 2). Apart from minor differences in structural MRI scanning parameters (detailed below), study protocols and task procedures were consistent across both studies.

The sample was approximately 47.4% female, with the following ethnic distribution: 30.1% European American, 20.3% Multi-ethnic, 20.3% Hispanic/Latinx, 12% Asian American, 7.5% Other, 7.5% African American, and 0.4% Native American. Previous papers have focused on age differences in giving and activation in a priori neural regions of interest (Karan et al., 2022) and functional connectivity depending upon the amount and target of giving (Uy et al., 2023), but none have examined the role of values placed upon giving to others.

### 2.2 Procedure

Participants completed a costly giving task in the scanner that was previously adapted to investigate prosocial decision-making (Telzer et al., 2011, Telzer et al., 2013, Telzer et al., 2014, Telzer et al., 2015). Before being introduced to the task, participants selected a friend and a caregiver without knowing that they would later earn money for them. Participants also completed questionnaires, including a measure of family obligation values.

#### 2.2.1 Family obligation values

To evaluate participants' family obligation values, participants rated 25 items on a 5-point scale assessing their values toward (1) current assistance to the family, (2) respect for the family, and (3) future support to the family (Fuligni et al., 1999). The current assistance subscale is measured using a scale from 1 (Does Not Apply) to 5 (Almost Always) that measures participants' expectations regarding the frequency of helping with household tasks and spending time with family. Sample items included: “help take care of your brothers and sisters,” “eat meals with your family,” and “spend time with your family on weekends.” The respect for the family subscale is measured using a scale from 1 (Does Not Apply) to 5 (Very Important) that assesses beliefs about the importance of honoring and adhering to family members’ wishes, expectations, and desires, with items such as “make sacrifices for your family,” “respect your older brothers and sisters,” and “show great respect for your parents.” The future support for the family subscale is measured using a scale from 1 (Does Not Apply) to 5 (Very Important) that captures participants' views on the importance of providing assistance and staying close to their family in the future, including statements like “help your parents financially in the future,” “help take care of your brothers and sisters in the future,” and “have your parents live with you when you get older.”

All 25 items were averaged to create a composite family obligation values index, ranging from 1 to 5, with higher scores reflecting stronger family obligation values. Additionally, subscale scores were computed separately for current assistance, respect for the family, and future support for the family for follow-up analyses that are included in the Supplemental Information. The mean for the overall family obligation values index was 3.42 (*SD* = 0.57, α =.81). The means for the subscales were as follows: future support for family, 3.29 (*SD* = 0.77, α =.80); respect for family, 3.80 (*SD* = 0.73, α =.83); and current assistance, 3.27 (*SD* = 0.68, α =.79). There were no significant age differences in family obligation in this sample (*b* =.03, *p* < .08).

#### 2.2.2 Giving task paradigm

Participants considered a series of financial offers that allowed them to earn money for themselves, their chosen caregiver and friend, as well as an unknown future participant (stranger). The task consisted of three runs, each dedicated to one of the three target recipients (caregiver, friend, or stranger), with the order of targets counterbalanced across participants. During each run, participants encountered four types of offers: (1) costly giving (40 trials per target), where the recipient gained money at a personal cost to the participant (e.g., YOU -$1.00, OTHER +$3.00); (2) non-costly reward (16 trials per target), where the participant earned money without affecting the recipient’s total (e.g., YOU +$3.00, OTHER -$0.00); (3) non-costly giving (five trials per target), where the recipient earned money at no cost to the participant (e.g., YOU -$0.00, OTHER +$3.00); and (4) control trials (16 trials per target), where neither the participant nor the recipient gained or lost money (e.g., YOU -$0.00, OTHER -$0.00).Using a handheld button box, participants indicated whether they accepted or rejected each offer. They were informed that a subset of trials would be randomly selected at the end of the task to determine the final earnings for themselves and their recipients.

**Fig. 1:**
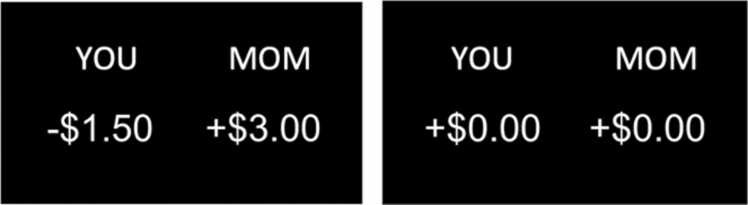
Example of a Costly Giving (left) and Control (right) trial. The specific target of the task, (caregiver, friend, or stranger) was indicated at the top right hand side.

The study compared costly giving trials to control trials presented within the same run, ensuring that visual and motor demands remained consistent (Fig. 1). Accepted costly giving trials were used as an index of giving behavior. Costly giving behavior was computed as the number of trials that participants accepted divided by the number of trials participants responded to (responses of accept and reject only). Trials that the participant did not respond to were excluded from the denominator. Additional trial types were included to introduce variability in decision-making and maintain participant engagement throughout the task. Offer values ranged in $0.25 increments, with participant earnings varying from –$3.75 to $0 and from + $2.00 to + $7.00, while recipients could receive between $0 and + $7.00. In every costly giving trial, the recipient’s potential gain exceeded the participant’s cost. Each offer was displayed for 3 s, during which participants could choose to accept or reject it, followed by a jittered fixation (500–4000 ms). At the end of the task, participants were paid in cash based on earnings from 10 randomly selected trials. Their payouts included personal earnings and the amount allocated to their friend and caregiver. Additionally, participants received an amount designated by the previous participant for their "stranger" recipient, while their own stranger earnings were passed on to the next participant in the study.

### 2.3 Functional MRI data preprocessing and analysis

Neuroimaging data were collected using a Siemens Prisma 3-Tesla MRI scanner at UCLA’s Staglin International Mental Health Research Organization Center for Cognitive Neuroscience. To minimize head movement and enhance comfort, foam padding was placed around each participant’s head. The task was displayed via a projector and viewed through a mirror mounted on the head coil.

For each participant, an initial set of three (one in each plane: coronal, sagittal, axial) 2D structural scout (localizer) gradient-echo images (repetition time [TR]=3.15 ms, echo time [TE]=1.37 ms, matrix size = 160 × 160, FoV=260 mm, 128 slices, flip angle=8°, 1.6-mm thick, 1.6-mm inplane resolution, 0.32-mm gap) was acquired in order to enable prescription of slices obtained in structural and functional scans. A T1-weighted magnetization prepared rapid gradient echo (MPRAGE) structural scan (parameters for participants from Study 1: TR=1900 ms, TE=2.26 ms, matrix size = 256 × 256, FoV=250 mm, 176 slices, flip angle=9°, 1-mm thick, 1-mm inplane resolution, 0.5-mm gap; parameters for participants from Study 2: TR=2000 ms, TE=2.52 ms, matrix size = 256 × 256, FoV=256 mm, 192 slices, flip angle=12°, 1-mm thick, 1-mm inplane resolution, 0.5-mm gap), coplanar with the functional scans, was collected for all participants.

The giving task consisted of three functional (echo planar T2*-weighted gradient-echo) MRI scans. Each functional run (TR=2000 ms, TE=30 ms, matrix size = 64 × 64, FoV=192 mm, 34 slices, flip angle=90°, 4-mm thick, 3-mm inplane resolution, no gap) lasted 6 min and 40 s.

### 2.4 fMRI data preprocessing and analysis

#### 2.4.1 fMRI data preprocessing

fMRI data were preprocessed and analyzed using the FMRIB Software Library (FSL version 5.0). Preprocessing for each run included skull-stripping, motion correction, slice timing correction, nonlinear high-pass temporal filtering (128 s), and spatial smoothing (6-mm FWHM). Functional images were registered to the high-resolution MPRAGE (6 ° of freedom) and then to standard Montreal Neurological Institute space (12 ° of freedom).

#### 2.4.2 Definition of regions of interest

For ROI activation, we selected an anatomically defined bilateral nucleus accumbens (NAcc) as the seed region of interest (ROI) that is part of the ventral striatum (VS) and was derived from the Harvard-Oxford atlas, thresholded at 50%. For the psychophysiological interaction (PPI) analyses, we selected the bilateral VS as the seed, which was defined by combining the caudate and putamen from the AAL atlas and was constrained within the coordinates −24 < x < 24, 4 < y < 18, and −12 < z < 0, following previous research (Inagaki and Eisenberger, 2012). To examine connectivity with the VS, we selected a set of a priori ROIs associated with cognitive control and social cognition. ROIs related to cognitive control included the bilateral dorsolateral prefrontal cortex (dlPFC) and ventrolateral prefrontal cortex (vlPFC). The dlPFC ROI was anatomically defined based on Broadman’s areas using the Wake Forest University (WFU) PickAtlas (Maldjian et al., 2003), while the bilateral vlPFC ROI was defined using the Harvard-Oxford Cortical Structural Atlas probability map in FSL, thresholded at 25% probability. ROIs associated with social cognition included the bilateral temporoparietal junction (TPJ), dorsomedial prefrontal cortex (dmPFC), and posterior superior temporal sulcus (pSTS). The TPJ ROI was created by combining the right TPJ (2812 voxels, all z > 6 mm, centered at [54, −52, 23]) and the left TPJ (2444 voxels, all z > 6 mm, centered at [−52, −58, 25]), following Dufour et al. (2013). The dmPFC ROI was defined using Neurosynth by searching and extracting the dmPFC region from the automated meta-analysis tool and masking it with the medial frontal gyrus from the WFU PickAtlas, based on prior work (Maldjian et al., 2003, Yarkoni et al., 2011). The pSTS ROI was created by extending the Desikan-Killiany Atlas-defined (Desikan et al., 2006) bank of the superior temporal sulcus to the border of the TPJ, following Mills et al. (2014). The dlPFC, TPJ, and dmPFC ROIs were based on work by Telzer and colleagues, available on NeuroVault (https://neurovault.org/collections/SISNGRAB/).

#### 2.4.3 fMRI data analysis

Following preprocessing, a general linear model was conducted for each individual run (target). Trials in which offers were accepted for each condition were modeled in separate regressors so they could be separately examined. Control trials were modeled in a single regressor, regardless of whether the trial was accepted or rejected, given that the financial outcome in these trials was identical. A linear contrast comparing accepted costly giving trials to control trials within the same run was computed for each participant in order to examine prosocial behavior. To examine corticostriatal connectivity, we conducted PPI analyses. For each run, we extracted the deconvolved time-series from the VS seed region (physiological regressor), convolved accepted costly giving versus control trials with the canonical double-gamma hemodynamic response function (psychological regressor), and multiplied the time-series of the physiological regressor by the psychological regressor (psychophysiological interaction term). This term identifies the ROIs we selected (dlPFC, vlPFC, dmPFC, pSTS, TPJ) that co-vary with the VS as a function of costly giving. Other explanatory variables—non-costly reward, non-costly giving, and control trials—were modeled using stick functions convolved with the canonical double-gamma hemodynamic response function. Mean activation values for the ROIs were also extracted; however, no significant associations were found with family obligation values. As such, these results are not discussed here but are included in the Supplemental Information.

### 2.5 Analysis plan

Linear mixed effects models were analyzed in R using the lme4 package to estimate giving behavior, ROI activation and VS connectivity with the other ROIs during accepted costly giving versus control trials as a function of target (family as reference group, friend, stranger), age of the giver, and family obligation values, which were mean centered. Costly giving behavior was computed as a percentage of accepted trials, such that the number of trials that participants accepted was divided by the number of trials participants responded to (specifically accepted and rejected responses). For each VS connectivity outcome, a model was run with the predictor being a three-way interaction term (family obligation x target x age) and all lower order terms. For behavioral outcomes (denoted by percentage of accepted costly giving trials), a model was run with the predictor being a 3-way interaction term (family obligation x target x age). In order to account for multiple comparisons, a Bonferroni correction was used to account for the ROI analysis of 6 separate ROIs and 5 for the functional connectivity analyses. With a family-wise error rate of p < .05, effects from the connectivity models had to be p < .01 in order to achieve statistical significance. All models were re-run to control for sex and ethnicity. Given that none of these covariates were found to be associated with the outcome variables, results are reported from models that exclude these covariates.

## 3 Results

### 3.1 Behavioral results

A sense of family obligation did not predict costly giving behavior to the family (see Table 1). There also were no significant interactions between family obligation and target or age in predicting costly giving. As previously reported in Karan et al. (2022), individuals reported greater costly giving towards family as compared to friends and strangers. Giving to family and friends increased with age, but age-related giving to strangers is reduced.

**Table 1:** Results from mixed-effects models estimating costly giving behavior during accepted costly donation trials as a function of family obligation values, target, and age.

	**Costly Giving Behavior**		
*Predictors*	*Estimates*	*SE*	*p*
Intercept	0.540	0.019	**< 0.001**
Friend	−0.081	0.016	**< 0.001**
Other	−0.168	0.016	**< 0.001**
Family Obligation	−0.001	0.033	0.966
Age	0.011	0.006	0.060
Family Obligation x Friend	−0.017	0.028	0.544
Family Obligation × Other	−0.050	0.028	0.069
Friend x Age	−0.001	0.005	0.879
Other x Age	−0.012	0.005	**0.010**
Family Obligation × Age	0.005	0.009	0.600
Family Obligation × Friend × Age	−0.014	0.008	0.072
Family Obligation × Other × Age	−0.002	0.008	0.755

### 3.2 Functional connectivity analyses

Family obligation predicted functional connectivity between the VS and three of the modeled ROIs (dmPFC, pSTS, TPJ), but the nature of the associations differed by target and age. As shown in Table 2, significant Family Obligation x Target (Family) x Age interactions were observed for VS-dmPFC, VS-pSTS, and VS-TPJ connectivity. In contrast, the nature of the association of family obligation with functional connectivity while giving to family and friends remains similar across ages.

**Table 2:** Results from mixed-effects models estimating functional connectivity between vs and social-cognitive and cognitive control ROIs during accepted costly donation trials as a function of family obligation values, target, and age.

	**VS-dmPFC Connectivity**			**VS-pSTS Connectivity**			**VS-TPJ Connectivity**			**VS-dlPFC Connectivity**			**VS-vlPFC Connectivity**		
*Predictors*	*Estimates*	*SE*	*p*	*Estimates*	*SE*	*p*	*Estimates*	*SE*	*p*	*Estimates*	*SE*	*p*	*Estimates*	*SE*	*p*
Intercept	−0.213	0.256	0.405	−0.211	0.204	0.303	−0.266	0.206	0.197	−0.058	0.184	0.751	0.081	0.192	0.672
Family Obligation	0.536	0.456	0.241	0.022	0.364	0.951	−0.005	0.367	0.989	−0.186	0.327	0.570	−0.392	0.342	0.253
Friend	0.092	0.413	0.824	0.001	0.337	0.998	0.165	0.341	0.629	−0.067	0.304	0.824	−0.257	0.317	0.419
Other	0.060	0.350	0.864	0.384	0.288	0.183	0.365	0.291	0.211	0.315	0.259	0.224	0.111	0.271	0.683
Age	0.032	0.076	0.671	−0.089	0.061	0.147	−0.037	0.062	0.548	−0.046	0.055	0.398	−0.080	0.057	0.163
Family Obligation x Friend	−1.036	0.740	0.162	0.524	0.605	0.386	0.249	0.611	0.684	0.227	0.544	0.677	0.459	0.569	0.420
Family Obligation × Other	0.341	0.623	0.585	0.052	0.513	0.919	0.297	0.518	0.566	0.312	0.461	0.499	0.627	0.482	0.194
Family Obligation × Age	−0.388	0.128	0.003	−0.295	0.102	0.004	−0.290	0.103	0.005	−0.199	0.092	0.030	−0.161	0.096	0.093
Friend x Age	−0.016	0.120	0.896	0.127	0.098	0.194	0.067	0.099	0.495	0.060	0.088	0.493	0.079	0.092	0.391
Other x Age	0.033	0.105	0.752	0.146	0.086	0.090	0.056	0.087	0.519	0.119	0.077	0.125	0.175	0.081	0.031
Family Obligation × Friend × Age	0.423	0.193	0.028	0.264	0.158	0.095	0.284	0.159	0.075	0.246	0.142	0.083	0.228	0.148	0.126
Family Obligation × Other × Age	0.384	0.175	0.028	0.317	0.144	0.028	0.332	0.145	0.022	0.226	0.130	0.081	0.225	0.135	0.097

**NOTE.** Family Obligation was mean-centered at 3.4 and Age was mean-centered at 13 years old. Family Obligation x Age indicates the interaction Family Obligation x Connectivity when giving to the Family x Age.

**Fig. 2:**
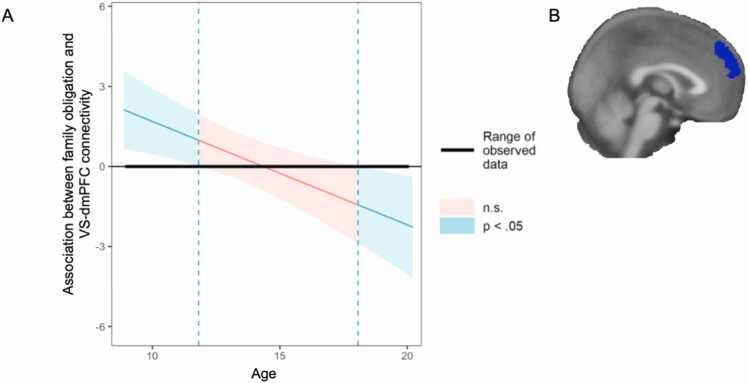
(A) The slope of family obligation on VS-dmPFC functional connectivity when giving to the family is significantly positive for adolescents between the ages 9–12 years old and significantly negative for adolescents between the ages 18–20 years old. (B) dmPFC brain map.

**Fig. 3:**
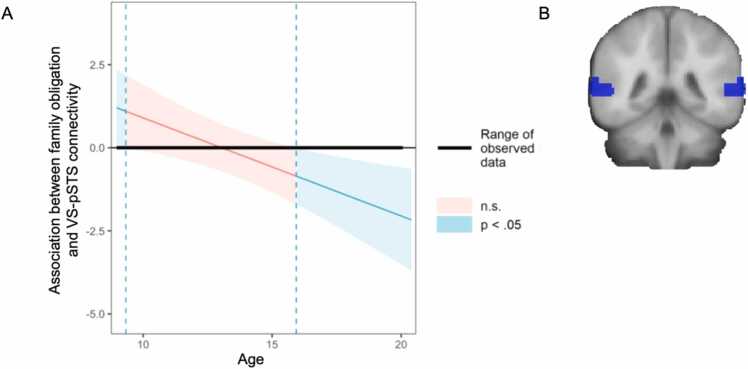
(A) The slope of family obligation on VS-pSTS functional connectivity when giving to the family is only significantly negative for adolescents approximately between the ages 16–20 years old. (B) dmPFC brain map.

**Fig. 4:**
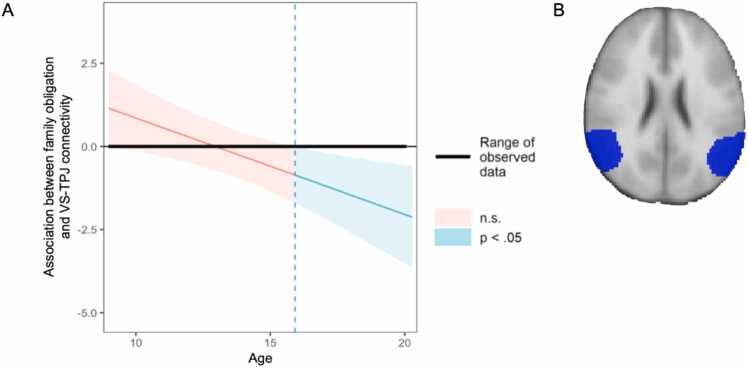
(A) The slope of family obligation on VS-TPJ functional connectivity when giving to the family is only significantly negative for adolescents approximately between the ages 16–20 years old. (B) pSTS brain map.

**Fig. 5:**
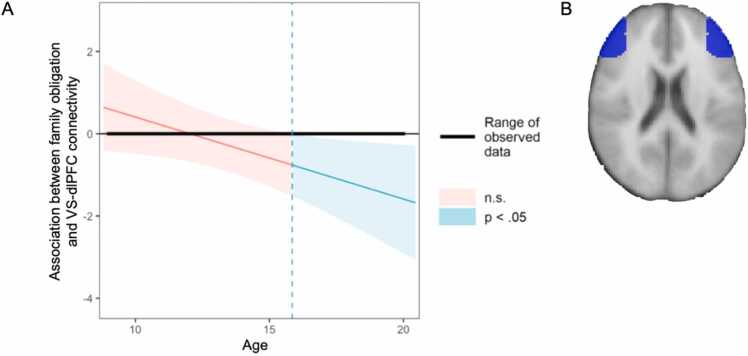
(A) The slope of family obligation on VS-dlPFC functional connectivity when giving to the family is only marginally significantly negative for adolescents approximately between the ages 16–20 years old. (B) dlPFC map.

Fig. 2, Fig. 3, Fig. 4 demonstrate that a greater sense of family obligation predicted stronger VS-dmPFC functional connectivity for younger ages when giving to the family, but that association changed such that across age that family obligation predicted less VS-dmPFC, -pSTS, and -TPJ functional connectivity among older adolescents. Following connectivity analyses, we conducted the Johnson-Neyman technique, which specifies the specific ages at which the slopes of family obligation values on functional connectivity are significantly positive and significantly negative. Thus, Fig. 2 specifically shows that the slope of family obligation on VS-dmPFC functional connectivity is significantly positive for adolescents approximately between the ages 9–12 years old and significantly negative for adolescents approximately between the ages 18–20 years old. Whereas, Fig. 3, Fig. 4 show that this estimated association between family obligation on VS-pSTS and VS-TPJ functional connectivity is not significant for younger ages, but there is a significant negative association for adolescents approximately between the ages 16–20 years old. The Family Obligation x Age interaction predicted VS-dlPFC connectivity while giving to the family at a trending level (see Table 2). As shown in Fig. 5A, greater emphasis placed on family obligation only predicted weaker connectivity for older adolescents.

Associations examining variation between individual family obligation values subscales and coupling in functional networks were generally similar to those observed with the overall scale and are included in the Supplemental Information.

## 4 Discussion

Value-based decision-making is a complex, dynamic process shaped by social experiences, particularly with close others such as family (Calderón-Tena et al., 2011). The current study examined how functional connectivity between networks supporting reward, social cognition, and cognitive control during prosocial giving varies as a function of family obligation values, age, and target recipient (family, friend, stranger). Results demonstrated that family obligation values consistently predicted negative functional connectivity between the VS and these key networks during decisions to give to the family for older adolescents and this association was sometimes positive at younger ages. These patterns reflect functional coupling associated with the context of value-based decisions, rather than formally derived neural value signals. We found limited evidence that family obligation values predicted functional connectivity during decisions to give to friends and strangers, suggesting that the value-based functional coupling linked with family obligation among older adolescents is specific to giving to only the family. In contrast, we did not find differences in giving behavior nor regional activation as a function of family obligation values, highlighting the importance of functional connectivity in the context of values and prosocial decision-making towards others during adolescence.

Our findings suggest that these neural circuits are involved in evaluating the value of prosocial decisions to the family and change with age, which may also be consistent with prior work showing that subcortical circuits such as the VS support behavior earlier in development and shape cortical maturation into young adulthood (Casey et al., 2019). Converging neuroeconomic accounts of value-based decision-making assert that affective and motivational processes involve subcortical regions such as the VS that integrate signals from higher-order cortical regions (e.g., mPFC) for value computation, which then yields motivated behavior (Insel et al., 2020, Pfeifer and Berkman, 2018, Samanez-Larkin and Knutson, 2015). Importantly, our findings may emphasize that the heterogeneity in individuals’ subjective weight or value placed on a familial obligation is then transformed into a common neural currency in the brain (Pfeifer and Berkman, 2018, Rangel and Hare, 2010). That this association between family obligation values consistently predicted connectivity when making value-based decisions demonstrates a potential mechanism for top-down modulation of the subcortical input to reflect value representation in adolescent decision-making (Casey et al., 2015, Hartley and Somerville, 2015).

Our findings demonstrate that the family obligation value-based functional connectivity between the VS and mentalizing- and cognitive control-related regions differed according to age. Specifically, greater value placed on family obligation predicted stronger functional connectivity between the VS and dmPFC for younger adolescents approximately between the ages 9–12 when giving to the family, but that association become negatively correlated across age among older adolescents approximately between the ages 18–20. Relatedly, this association for lower VS coupling with the pSTS and TPJ was only significant after age 16 (and marginally significant with the dlPFC). For younger individuals who strongly value helping their family, stronger connectivity between the VS and dmPFC may reflect the fact that decisions favoring greater reward outcomes for the family involves greater engagement in the VS, which then upregulates activation in the dmPFC toward the outcomes of giving to the family, thereby promoting a better understanding of the perspectives of the family. Indeed, earlier research has found that gaining money for family members as compared with a stranger was associated with increased activation in the nucleus accumbens, a region located within the VS, and mentalizing regions such as the mPFC, possibly demonstrating increased value and mentalizing computations when giving to the family (Braams and Crone, 2017, Brandner et al., 2021, Morelli et al., 2015, Spaans et al., 2018). The observed connectivity between these regions at younger ages may reflect the need for greater coordination with sociocognitive processes in order to elicit these prosocial decisions towards the family (Amodio and Frith, 2006, Blakemore, 2008, Knight et al., 2015, Telzer et al., 2011). Whereas with older adolescents, the observed negative functional coupling between frontostriatal networks might demonstrate that these systems still work in tandem together, but in opposite directions, in order to elicit giving to the family at the neurobiological level. Behavioral work suggests that while initial decreases in prosocial behavior toward family members are typical during early adolescence, a rebound likely occurs during middle to late adolescence (Padilla-Walker et al., 2018). The decoupling between the striatum and mentalizing and cognitive control regions during decisions to help the family might be related to later maturational processes (e.g., more sophisticated understanding of reasons to help the family), from greater expectations from parents placed upon older adolescents, or from changes in the parent–child relationship during late adolescence and the transition to adulthood (Calderón-Tena et al., 2011). As such, the observed negative frontostriatal connectivity might reflect the engagement of mentalizing and cognitive control regions to exert increased top-down regulation over the VS, potentially driven by a heightened sense of familial obligation during prosocial decision-making. This observation may be consistent with work demonstrating that late development of the prefrontal cortex and continued development of corticostriatal connectivity may influence value-based decision-making (Hartley and Somerville, 2015, Insel et al., 2017). Older adolescents with strong family obligation values may be engaging in qualitatively different cognitive processes (e.g., maintaining social norms and preferences in memory when making these decisions), possibly suggesting that it becomes more of an automatic decision to help the family across development that isn’t necessarily reward-inducing as it may be for younger adolescents (Telzer et al., 2011). Indeed, our findings seem to be consistent with Kwon et al. (2022), in which they demonstrated that functional connectivity in these networks changes developmentally to support other-oriented decision-making, such that early in development, greater connectivity supports other-oriented decisions, whereas later in development, less connectivity supports other-oriented decisions. These findings highlight that mentalizing and cognitive control networks may differentially modulate the value placed on family obligation during value-based functional coupling in the VS at different developmental stages (Pfeifer and Berkman, 2018, Zaki and Mitchell, 2011).

Although previous research has found that corticostriatal regions are recruited more during costly giving to in-group relative to out-group members Hackel et al., 2017, Telzer et al., 2015, Zaki and Mitchell, 2011), we did not find evidence that youth exhibited differences in activation when giving to different targets. Rather, differences in age during decisions to help the family emerged when we examined functional connectivity. Further, while individuals reported greater giving towards the family relative to friends and strangers, family obligation values did not predict giving behavior. It is possible that functional connectivity changes to promote the same behavior. In other words, their behavior doesn't change as a function of family obligation; rather, the neural circuits that support this link change developmentally to support the same behavior. Our findings suggest that connectivity between neural systems involved in reward, mentalizing, and cognitive control, rather than regional activation–all of which undergo significant functional maturation in adolescence–could elucidate the complexity of prosocial decision-making (Casey et al., 2015). It is possible that the consistent patterns of connectivity between these regions might reflect a general corticostriatal value-based integration rather than multiple distinct mechanisms. Our findings may coincide with prior research showing that the value of the prosocial decision (e.g., giving value, greater discrepancy between potential rewards for others over oneself) has differential associations with functional connectivity between reward and mentalizing regions across adolescence (Baumgartner et al., 2015, Do and Telzer, 2019, Uy et al., 2023). These findings highlight the dynamic interaction between social-cognitive and reward-valuation regions to help adolescents differentiate the salience and reward associated with making prosocial decisions when the decisions affect their family.

These findings should be interpreted with consideration of the study’s limitations. Despite our giving task being well-validated across previous studies and facilitation of the inclusion of younger individuals, the simplicity of our task limited our ability to investigate more complex features of prosocial behavior, such as the need of the recipients, which may involve greater mentalizing or social cognition. We also do not have attitudinal measures towards friends to be able to compare differences between values for close others. Future studies would benefit from examining attitudinal measures toward friends and strangers to better assess whether similar value-based constructs operate across different social targets. Our analyses revealed age differences in functional connectivity in corticostriatal networks during prosocial decision-making; however, the cross-sectional nature of the study precludes conclusions regarding developmental change. Moreover, we did not have data on individuals between 16 and 18 years of age, so our age effects are only interpolated for this age group. It is possible that there may be non-linearity for these particular ages that we were unable to estimate. Empirical findings on the development of prosocial behaviors during adolescence are relatively inconclusive, with documented age-related increases (Güroğlu et al., 2014, van de Groep et al., 2022) and stabilization (Padilla-Walker et al., 2018) in prosocial decisions to the family. In previous analyses using the same dataset, researchers found age-related increases in connectivity as giving value increased when considering giving to known others (Uy et al., 2023). Future studies that utilize longitudinal approaches and extend into young adulthood would help to clarify the differences in functional connectivity for younger versus older adolescents, given the continued maturation of key brain networks involved in prosocial behavior. Family obligation values were also used as a proxy for “value” when considering making prosocial decisions to multiple recipients and associated functional connectivity.

Taken together, the current study provides novel evidence for the consideration of family obligation values being represented as “value” in the developing brain during decisions to help the family across adolescence. To our knowledge, this is one of the first studies to examine how other-oriented values interact with brain development in shaping adolescent prosociality (Telzer et al., 2011, Yang et al., 2024). The decision to help others is rather complex, drawing on multiple social and contextual motives and requires the integration of cognitive and affective processes including valuation, self-control, and social cognition (Amodio and Frith, 2006, Crone et al., 2022, Crone and Fuligni, 2020, Lieberman et al., 2019). We demonstrate that the degree to which individuals value helping the family shows differentiated functional connectivity between the VS and mentalizing- and cognitive control-related regions when giving to the family during adolescence, showing that it may be family-specific, rather than tapping prosocial behavior toward others more generally. The shift in functional coupling may underlie changing sensitivity to reward in the context of decision-making for the self and others (Crone and Fuligni, 2020). Our findings underscore the importance of utilizing functional connectivity approaches to grasp the complexities when considering the value placed on helping the family across development. Our study also extends the large body of social neuroeconomic research by exploring values that may orient individuals towards helping others. We highlight the important role of reward, mentalizing, and cognitive control networks in developmental changes associated with family obligation values, especially when considering implications for increasing out-group prosocial behaviors. Importantly, our study highlights the need to incorporate social and cultural context into neurocognitive models of prosocial development and to identify the multiple neural pathways through which values guide behavior towards family and broader social groups.

## CRediT authorship contribution statement

**Jasmine Hernández:** Writing – review & editing, Writing – original draft, Visualization, Software, Methodology, Investigation, Formal analysis, Data curation, Conceptualization. **Jessica Uy:** Writing – review & editing, Software, Methodology, Investigation, Data curation. **Eva H. Telzer:** Writing – review & editing. **Andrew J. Fuligni:** Writing – review & editing, Writing – original draft, Supervision, Resources, Project administration, Methodology, Funding acquisition, Conceptualization. **Naomi I. Eisenberger:** Writing – review & editing, Supervision, Resources, Project administration, Methodology, Funding acquisition, Conceptualization. **Adriana Galván:** Writing – review & editing, Writing – original draft, Supervision, Resources, Project administration, Methodology, Funding acquisition, Conceptualization.

## Funding

This research was supported by the National Institute of Child Health and Human Development (NICHD) (#5T32HD091059) awarded to J.H, the National Science Foundation Award (#1551952) to A.J.F., N.I.E., and A.G., and the 10.13039/100000071NICHD (#1R01HD093823–01) awarded to A.J.F., N.I.E., and A.G.

## Declaration of Competing Interest

The authors declare that they have no known competing financial interests or personal relationships that could have appeared to influence the work reported in this paper.

## Data Availability

Data will be made available on request.
