# Supplementary material for: Neural representations of the value of helping the family during adolescence
Source: Dev Cogn Neurosci. 2026 Jun 27;80:101770. doi: 10.1016/j.dcn.2026.101770 (PMC13352427; doi:10.1016/j.dcn.2026.101770)
Supplement: Supplementary file 1 — Supplementary material [file mmc1.docx]

Supplemental Information

**Table S1.** Results from Mixed Effects Models Estimating ROI Activation During Accepted Costly Donation Trials as a Function of Family Obligation Values, Target, and Age. A sense of family obligation did not predict activation in any of the target ROIs during accepted giving trials relative to control trials. There also were no significant interactions between family obligation and target or age in predicting ROI activation.


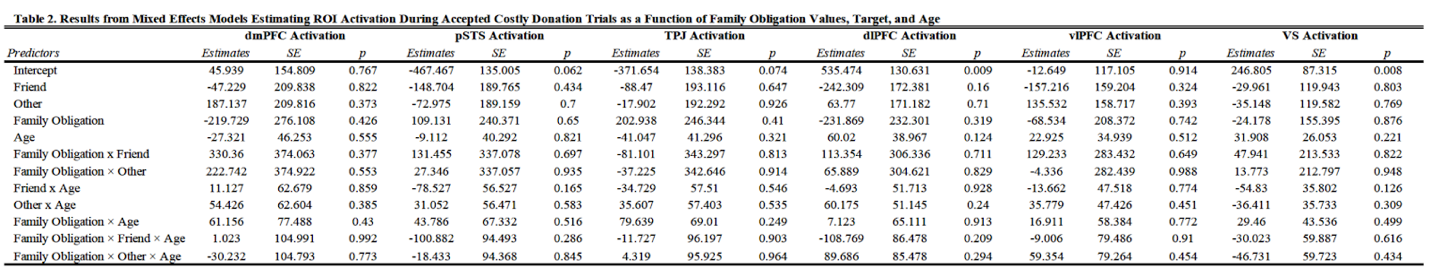


| **Table S2.**  Results from Mixed-Effects Models Estimating Functional Connectivity between VS and Cognitive Control ROIs During Accepted Costly Donation Trials as a Function of Family Obligation Value Subscales, Target, and Age. | | | | | | |
| --- | --- | --- | --- | --- | --- | --- |
|  | ***VS-dlPFC Connectivity*** | | | ***VS-vlPFC Connectivity*** | | |
| *Predictors* | *Estimates* | *SE* | *p* | *Estimates* | *SE* | *p* |
| Intercept | -0.033 | 0.183 | 0.858 |  |  |  |
| Future Support | 0.134 | 0.241 | 0.578 |  |  |  |
| Target (Friend) | -0.128 | 0.302 | 0.671 |  |  |  |
| Target (Other) | 0.266 | 0.258 | 0.304 |  |  |  |
| Age | -0.021 | 0.055 | 0.7 |  |  |  |
| Future Support × Target (Friend) | -0.07 | 0.413 | 0.866 |  |  |  |
| Future Support × Target (Other) | -0.237 | 0.341 | 0.487 |  |  |  |
| Future Support × Age | -0.134 | 0.068 | **0.049*** |  |  |  |
| Target (Friend) × Age | 0.031 | 0.087 | 0.722 |  |  |  |
| Target (Other) × Age | 0.087 | 0.077 | 0.26 |  |  |  |
| Future Support × Target (Friend) × Age | 0.104 | 0.111 | 0.349 |  |  |  |
| Future Support × Target (Other) × Age | 0.099 | 0.096 | 0.301 |  |  |  |
| Intercept | -0.041 | 0.183 | 0.821 | 0.073 | 0.19 | 0.7 |
| Respect for Family | -0.089 | 0.249 | 0.72 | -0.059 | 0.259 | 0.821 |
| Target (Friend) | -0.087 | 0.302 | 0.774 | -0.258 | 0.315 | 0.414 |
| Target (Other) | 0.313 | 0.258 | 0.226 | 0.129 | 0.269 | 0.63 |
| Age | -0.045 | 0.055 | 0.409 | -0.078 | 0.057 | 0.171 |
| Respect for Family × Target (Friend) | -0.206 | 0.417 | 0.622 | -0.139 | 0.434 | 0.749 |
| Respect for Family × Target (Other) | 0.138 | 0.351 | 0.694 | 0.253 | 0.366 | 0.49 |
| Respect for Family × Age | -0.156 | 0.072 | **0.032*** | -0.205 | 0.076 | **0.007*** |
| Target (Friend) × Age | 0.05 | 0.088 | 0.572 | 0.068 | 0.091 | 0.454 |
| Target (Other) × Age | 0.119 | 0.077 | 0.122 | 0.176 | 0.08 | **0.029*** |
| Respect for Family × Target (Friend) × Age | 0.219 | 0.117 | **0.061** | 0.268 | 0.122 | **0.028*** |
| Respect for Family × Target (Other) × Age | 0.229 | 0.102 | **0.026*** | 0.305 | 0.107 | **0.004*** |
| Intercept | -0.034 | 0.24 | 0.887 | 0.128 | 0.229 | 0.576 |
| Current Assistance | -0.342 | 0.355 | 0.335 | -0.523 | 0.338 | 0.122 |
| Target (Friend) | 0.322 | 0.327 | 0.325 | -0.038 | 0.321 | 0.904 |
| Target (Other) | 0.289 | 0.326 | 0.376 | 0.051 | 0.320 | 0.871 |
| Age | -0.047 | 0.072 | 0.516 | -0.072 | 0.068 | 0.294 |
| Current Assistance × Target (Friend) | 1.096 | 0.481 | **0.023*** | 1.144 | 0.472 | **0.016*** |
| Current Assistance × Target (Other) | 0.549 | 0.48 | 0.253 | 0.677 | 0.471 | 0.151 |
| Current Assistance × Age | -0.103 | 0.1 | 0.304 | -0.027 | 0.095 | 0.77 |
| Target (Friend) × Age | 0.024 | 0.098 | 0.808 | 0.033 | 0.096 | 0.725 |
| Target (Other) × Age | 0.123 | 0.098 | 0.208 | 0.165 | 0.096 | 0.086 |
| Current Assistance × Target (Friend) × Age | 0.046 | 0.136 | 0.734 | 0.001 | 0.133 | 0.991 |
| Current Assistance × Target (Other) × Age | 0.122 | 0.136 | 0.37 | 0.052 | 0.133 | 0.696 |
| Intercept | -0.058 | 0.184 | 0.751 |  |  |  |
| Overall Family Obligation | -0.186 | 0.327 | 0.57 |  |  |  |
| Target (Friend) | -0.067 | 0.304 | 0.824 |  |  |  |
| Target (Other) | 0.315 | 0.259 | 0.224 |  |  |  |
| Age | -0.046 | 0.055 | 0.398 |  |  |  |
| Overall Family Obligation × Target (Friend) | 0.227 | 0.544 | 0.677 |  |  |  |
| Overall Family Obligation × Target (Other) | 0.312 | 0.461 | 0.499 |  |  |  |
| Overall Family Obligation × Age | -0.199 | 0.092 | **0.03*** |  |  |  |
| Target (Friend) × Age | 0.06 | 0.088 | 0.493 |  |  |  |
| Target (Other) × Age | 0.119 | 0.077 | 0.125 |  |  |  |
| Overall Family Obligation × Target (Friend) × Age | 0.246 | 0.142 | 0.083 |  |  |  |
| Overall Family Obligation × Target (Other) × Age | 0.226 | 0.13 | 0.081 |  |  |  |
|  |  | | |  | | |
|  |  | | |  | | |

| **Table S3.** Results from Mixed-Effects Models Estimating Functional Connectivity between VS and Sociocognitive ROIs During Accepted Costly Donation Trials as a Function of Family Obligation Values, Target, and Age | | | | | | | | | |
| --- | --- | --- | --- | --- | --- | --- | --- | --- | --- |
|  | ***VS-dmPFC Connectivity*** | | | ***VS-pSTS Connectivity*** | | | ***VS-TPJ Connectivity*** | | |
| *Predictors* | *Estimates* | *SE* | *p* | *Estimates* | *SE* | *p* | *Estimates* | *SE* | *p* |
| Intercept | -0.144 | 0.255 | 0.571 | -0.172 | 0.203 | 0.397 | -0.22 | 0.206 | 0.286 |
| Future Support | 0.793 | 0.336 | **0.019** | 0.067 | 0.268 | 0.802 | 0.173 | 0.271 | 0.522 |
| Target (Friend) | 0.065 | 0.412 | 0.875 | -0.062 | 0.336 | 0.853 | 0.115 | 0.34 | 0.736 |
| Target (Other) | -0.048 | 0.349 | 0.892 | 0.342 | 0.287 | 0.233 | 0.291 | 0.29 | 0.317 |
| Age | 0.069 | 0.076 | 0.367 | -0.064 | 0.061 | 0.29 | -0.01 | 0.061 | 0.871 |
| Future Support × Target (Friend) | -1.186 | 0.563 | **0.036*** | 0.466 | 0.459 | 0.31 | 0.1 | 0.464 | 0.83 |
| Future Support × Target (Other) | -0.358 | 0.461 | 0.438 | -0.152 | 0.379 | 0.689 | -0.089 | 0.383 | 0.816 |
| Future Support × Age | -0.205 | 0.094 | **0.03*** | -0.178 | 0.075 | **0.018*** | -0.156 | 0.076 | **0.042*** |
| Target (Friend) × Age | -0.051 | 0.118 | 0.665 | 0.105 | 0.097 | 0.278 | 0.042 | 0.098 | 0.671 |
| Target (Other) × Age | -0.012 | 0.104 | 0.907 | 0.115 | 0.086 | 0.178 | 0.022 | 0.087 | 0.801 |
| Future Support × Target (Friend) × Age | 0.293 | 0.15 | **0.052** | 0.119 | 0.123 | 0.335 | 0.152 | 0.124 | 0.223 |
| Future Support × Target (Other) × Age | 0.093 | 0.13 | 0.476 | 0.196 | 0.107 | **0.066** | 0.121 | 0.108 | 0.262 |
| Intercept | -0.19 | 0.255 | 0.458 | -0.201 | 0.202 | 0.322 | -0.25 | 0.205 | 0.222 |
| Respect for Family | 0.181 | 0.347 | 0.603 | 0.199 | 0.275 | 0.47 | 0.066 | 0.279 | 0.813 |
| Target (Friend) | 0.054 | 0.411 | 0.895 | -0.031 | 0.335 | 0.925 | 0.141 | 0.339 | 0.678 |
| Target (Other) | 0.044 | 0.348 | 0.9 | 0.389 | 0.285 | 0.174 | 0.36 | 0.289 | 0.213 |
| Age | 0.018 | 0.076 | 0.811 | -0.09 | 0.06 | 0.137 | -0.039 | 0.061 | 0.521 |
| Target (Friend) × Age | -0.006 | 0.119 | 0.959 | 0.115 | 0.097 | 0.238 | 0.06 | 0.098 | 0.541 |
| Target (Other) × Age | 0.038 | 0.104 | 0.713 | 0.158 | 0.085 | 0.065 | 0.059 | 0.086 | 0.495 |
| Respect for Family × Target (Friend) | -0.577 | 0.567 | 0.31 | 0.043 | 0.462 | 0.925 | -0.097 | 0.467 | 0.836 |
| Respect for Family × Target (Other) | 0.28 | 0.474 | 0.555 | 0.095 | 0.389 | 0.807 | 0.113 | 0.394 | 0.775 |
| Respect for Family × Age | -0.313 | 0.101 | **0.002*** | -0.28 | 0.08 | **0.001*** | -0.251 | 0.081 | **0.002*** |
| Respect for Family × Target (Friend) × Age | 0.301 | 0.159 | 0.059 | 0.236 | 0.129 | 0.068 | 0.254 | 0.131 | 0.053 |
| Respect for Family × Target (Other) × Age | 0.353 | 0.138 | **0.011*** | 0.341 | 0.113 | **0.003*** | 0.335 | 0.115 | **0.004*** |
| Intercept | -0.177 | 0.258 | 0.494 |  |  |  |  |  |  |
| Current Assistance | 0.062 | 0.38 | 0.871 |  |  |  |  |  |  |
| Target (Friend) | 0.048 | 0.413 | 0.907 |  |  |  |  |  |  |
| Target (Other) | 0.04 | 0.35 | 0.909 |  |  |  |  |  |  |
| Age | 0.021 | 0.077 | 0.784 |  |  |  |  |  |  |
| Target (Friend) × Age | 0.004 | 0.12 | 0.977 |  |  |  |  |  |  |
| Target (Other) × Age | 0.044 | 0.105 | 0.677 |  |  |  |  |  |  |
| Current Assistance × Target (Friend) | -0.293 | 0.602 | 0.627 |  |  |  |  |  |  |
| Current Assistance × Target (Other) | 0.612 | 0.516 | 0.236 |  |  |  |  |  |  |
| Current Assistance × Age | -0.23 | 0.107 | **0.032*** |  |  |  |  |  |  |
| Current Assistance × Target (Friend) × Age | 0.241 | 0.162 | 0.137 |  |  |  |  |  |  |
| Current Assistance × Target (Other) × Age | 0.285 | 0.146 | **0.051*** |  |  |  |  |  |  |
| Intercept | -0.213 | 0.256 | 0.405 | -0.211 | 0.204 | 0.303 | -0.266 | 0.206 | 0.197 |
| Overall Family Obligation | 0.536 | 0.456 | 0.241 | 0.022 | 0.364 | 0.951 | -0.005 | 0.367 | 0.989 |
| Target (Friend) | 0.092 | 0.413 | 0.824 | 0.001 | 0.337 | 0.998 | 0.165 | 0.341 | 0.629 |
| Target (Other) | 0.06 | 0.35 | 0.864 | 0.384 | 0.288 | 0.183 | 0.365 | 0.291 | 0.211 |
| Age | 0.032 | 0.076 | 0.671 | -0.089 | 0.061 | 0.147 | -0.037 | 0.062 | 0.548 |
| Target (Friend) × Age | -0.016 | 0.12 | 0.896 | 0.127 | 0.098 | 0.194 | 0.067 | 0.099 | 0.495 |
| Target (Other) × Age | 0.033 | 0.105 | 0.752 | 0.146 | 0.086 | 0.09 | 0.056 | 0.087 | 0.519 |
| Overall Family Obligation × Target (Friend) | -1.036 | 0.74 | 0.162 | 0.524 | 0.605 | 0.386 | 0.249 | 0.611 | 0.684 |
| Overall Family Obligation × Target (Other) | 0.341 | 0.623 | 0.585 | 0.052 | 0.513 | 0.919 | 0.297 | 0.518 | 0.566 |
| Overall Family Obligation × Age | -0.388 | 0.128 | **0.003*** | -0.295 | 0.102 | **0.004*** | -0.29 | 0.103 | **0.005*** |
| Overall Family Obligation × Target (Friend) × Age | 0.423 | 0.193 | **0.028*** | 0.264 | 0.158 | 0.095 | 0.284 | 0.159 | 0.075 |
| Overall Family Obligation × Target (Other) × Age | 0.384 | 0.175 | **0.028*** | 0.317 | 0.144 | **0.028*** | 0.332 | 0.145 | **0.022*** |
|  |  | | |  | | |  | | |
